# Platelets and Complement Cross-Talk in Early Atherogenesis

**DOI:** 10.3389/fcvm.2019.00131

**Published:** 2019-09-06

**Authors:** Hugh Kim, Edward M. Conway

**Affiliations:** ^1^Centre for Blood Research, Life Sciences Institute, University of British Columbia, Vancouver, BC, Canada; ^2^Department of Biochemistry and Molecular Biology, University of British Columbia, Vancouver, BC, Canada; ^3^Faculty of Dentistry, University of British Columbia, Vancouver, BC, Canada; ^4^Faculty of Medicine, University of British Columbia, Vancouver, BC, Canada

**Keywords:** platelets, complement, atheroma, inflammation, atherosclerosis, endothelium, innate immunity, vascular

## Abstract

Atherosclerosis remains a ubiquitous and serious threat to human health. The initial formation of the atherosclerotic lesion (atheroma) is driven by pro-inflammatory signaling involving monocytes and vascular endothelial cells; later stages of the disease involve rupture of well-established atherosclerotic plaques, thrombosis, and blood vessel occlusion. While the central role of platelets in thrombosis is undisputed, platelets exhibit pro-inflammatory activities, and contribute to early-stage atheroma formation. Platelets also engage components of the complement system, an essential element of innate immunity that contributes to vascular inflammation. Here we provide an overview of the complex interplay between platelets and the complement system, with a focus on how the crosstalk between them may impact on the initiation of atheroma formation.

## Introduction

Cardio-cerebrovascular diseases are commonly caused by atherosclerosis, an inflammatory vasculopathy characterized by the formation of atheromatous plaques along the arterial wall. If the disease progresses, blood flow becomes limited, with resultant tissue injury, and organ dysfunction. Several pro-inflammatory events lead to plaque formation [reviewed in (1–3)]. Early in the process, endothelial activation promotes binding of monocytes to the vascular wall. These cells subsequently migrate into the subendothelial space, where they differentiate into macrophages. They then phagocytose oxidized low-density lipoproteins (oxLDL) which are deposited within the vascular wall, resulting in their transformation into lipid-laden foam cells. In parallel, vascular smooth muscle cells synthesize a fibrous connective tissue cap that surrounds the central lipid-macrophage core. Long-standing pro-coagulant and pro-inflammatory atherosclerotic plaques are prone to rupture, resulting in life-threatening thrombosis and ischemia, as is observed during myocardial infarction, and stroke. Platelets play a central role in thrombus formation secondary to plaque rupture. However, platelets are also highly immune-competent cells that, with activation of the complement system, are believed to orchestrate the initial signaling events during vascular inflammation that are critical for atheroma formation. In this review, we focus on the participation of platelet-complement crosstalk in early atherogenesis. We begin with a broad overview of the concepts of platelet activation and how this may trigger atheroma formation via interactions with leukocytes and endothelial cells [readers are referred to detailed reviews on this topic (4–6)]. This is followed by a description of the complement cascade and its regulation. We finally focus on the inter-relationship between platelets and the complement system, highlighting several of the complex links, how they may impact on atherogenesis, and on the potential clinical utility of recently uncovered pathways.

## Platelet Signaling Promotes Atherogenesis

### Platelet Activation and Secretion

Platelets are 2–4 μm diameter cells that circulate in resting discoid forms at a concentration of 150,000–450,000 cells/μl of whole blood, and become activated upon stimulation of their cell surface receptors by corresponding ligands (4). The role of platelets in thrombosis following plaque rupture in late-stage atherosclerosis is well-known. However, platelets also store >30 cytokines and growth factors in granules—alpha (α)-granules, dense granules, and lysosomes (7). Platelet activation is typically accompanied by rapid shape change, with fusion of the granule membranes with the platelet's plasma membrane, and the inner membranous network, to form the so-called open canalicular system (7, 8). Platelet secretion that occurs with this activation, is characterized by rapid translocation of P-selectin from the α-granules to the plasma membrane and extracellular release of soluble cytokines, chemokines, growth factors, and complement components (9). Activated platelets also uniquely express multiple receptors on their surface, and release platelet microparticles (PMPs), which are <1 μm diameter vesicular bodies containing a variety of cytokines, including for example, interleukin-1β (IL-1β (10). Importantly, platelet-derived cytokines from activated platelets are believed to contribute to vascular inflammation at the early stages of atheroma formation (11). Strong evidence exists that platelets also promote atherogenesis by acting as lipid-carrying structures (12), by signaling to vascular endothelium, and by recruiting leukocytes to the nascent atheroma (13).

### Platelet-Endothelial Cell Signaling

Under physiologic conditions, the endothelial cell layer is separated from circulating platelets and leukocytes by a proteoglycan-rich layer termed the glycocalyx (14). Endothelial cells also produce prostacyclin (15), and nitric oxide (16) which serve to maintain platelets in their quiescent states. However, activation of the endothelial cell layer promotes atheroma formation. Activated endothelial cells express platelet-adhesive molecules including for example, E-selectin (17) and von Willebrand factor (VWF) (18), and release platelet agonists such as thromboxane (19). These molecules serve to recruit, activate, and tether platelets to the vessel wall. The activated platelets in turn, express P-selectin (20), and release multiple cytokines and chemokines that further induce endothelial cell activation (21), with the result being more recruitment and activation of platelets at the site of the vascular lesion. Unless dampened by natural or pharmacologic interventions, the process becomes self-sustaining with progressive vascular damage, and atherogenesis.

### Platelet-Derived Chemokines Recruit and Retain Monocytes at Sites of Vascular Inflammation

In addition to promoting endothelial cell activation, platelet-derived chemokines recruit monocytes, thereby propagating atheroma development. CXCL4/PF4 (platelet factor four), a major constituent of platelet α-granules (22–25), is one of several well-characterized monocyte chemoattractants. CCL5/RANTES (regulated on activation normal T-cell expressed and secreted) is also a platelet-derived chemokine with monocyte chemoattractant properties (26, 27).

The pathophysiologic relevance of these and other platelet-released factors in atheroma formation is supported by several observations. For example, *in vitro* analyses revealed that monocytes preferentially adhere to endothelial cells pre-incubated with PF4 and/or RANTES (28, 29), while *in vivo* studies showed that the size of experimentally-induced atherosclerotic lesions were significantly reduced in PF4-null mice (30). Evidence also exists from clinical studies to support the contribution of PF4 and RANTES in atherosclerosis. In analyses of 132 carotid atheromatous plaques, PF4 presence directly correlated with lesion severity, in terms of histological grading of the lesions, and the history of significant clinical events such as myocardial infarction (31). RANTES plasma levels in patients hospitalized with acute coronary syndrome also correlated with progressive disease (32). That the chemotactic properties of PF4 and RANTES promote vascular disease severity underlines the importance of understanding how these and other similarly biologically active factors are released by platelets. Increasing evidence supports the notion that the complement system plays an important role.

## The Complement System

### Activation via 3 Pathways

The complement system is a tightly regulated blood borne proteolytic system, a key component of innate immunity, that responds rapidly to clear damaged host cells and invading pathogens, to limit tissue destruction and to effect healing. This system is intimately involved in platelet function and the pathogenesis and progression of atherosclerosis. Thus, a brief review is provided [more extensive reviews can be found in (33–36)].

**Figure 1:**
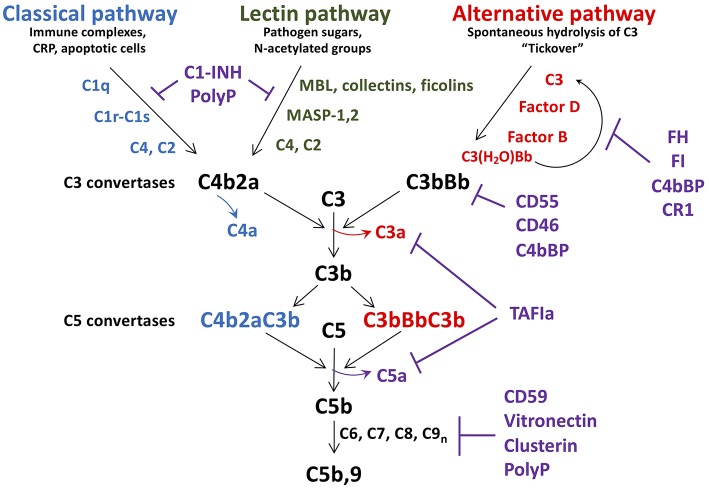
Schematic of complement activation and regulation. Complement activation occurs via the classical, lectin, or alternative pathways, triggered by exposure of C1q, MBL, collectins and ficolins, to danger signals. The alternative pathway is constitutively active, due to spontaneous hydrolysis of C3 to C3(H_2_O). All pathways converge to form C3 convertases, with release of C4a, and C3a. As C3b is further generated, C5 convertases C4bBbC3b, and C3bBbC3b are formed, resulting in release of the potent anaphylatoxin C5a, in concert with C5b. C5b is the initial factor required for assembly of the C5b-9 membrane attack complex which induces lysis/damage to the cellular target. Tight regulation is achieved at multiple levels by soluble and membrane associated factors (C1-INH, FH, FI, CD55, CD46, polyphosphate (polyP), CD59, clusterin, and vitronectin). C1-INH, C1 esterase inhibitor; MBL, mannose binding lectin; CRP, C-reactive protein; MASP, MBL associated serine protease; FH, factor H; FI, factor I; TAFIa, activated thrombin activatable fibrinolysis inhibitor.

Comprising over 30 soluble and membrane bound proteins, complement activation is triggered by exposure to damage-associated molecular patterns, initiated via the lectin (LP), classical (CP), or alternative (AP) pathways (Figure 1). The CP is triggered by C1q recognition of antibodies or other targets (e.g., C-reactive protein, apoptotic cells) bound to antigens or microbial surfaces. C1q circulates in complex with zymogen forms of serine proteases C1r and C1s. Exposure of C1q to its target results in activation of C1r and C1s (37), followed by C1s-mediated cleavage of C4 into C4a (an anaphylatoxin) and the opsonin C4b. C2 complexes with immobilized C4b, and is also cleaved by C1s into C2b and C2a. The resultant C4b2a complex is the CP C3 convertase which cleaves C3 into C3b, liberating the anaphylatoxin C3a. C3b binds to the surface of nearby cells/microbes for downstream complement activation.

Similar to the CP, in the LP, mannose binding lectin (MBL), ficolins, and/or collectin-11 circulate in complex with MBL-associated zymogens of serine proteases, MASP1/MASP3, and MASP2 (38). These complexes bind to sugars on micro-organisms or damaged cells, whereupon MASP1 autoactivates and cleaves C2, as well as MASP2, which then cleave C2 and C4, yielding C4b2a, the LP C3 convertase (39).

In contrast to the CP/LP, the AP is constitutively active via a “tick-over” mechanism in which small amounts of circulating C3 spontaneously hydrolyze into C3(H_2_O) (40). This yields a binding site for factor B (FB) which is cleaved into Ba and Bb by factor D. Bb binds to C3(H_2_O) to form a fluid-phase C3 convertase, which can cleave C3 to generate C3a and C3b. Relevant to a relationship between platelets and complement, surface contact may also trigger hydrolysis of C3 and thus activation of the AP (41). This is achieved via the release of properdin by inflammatory leukocytes, which binds to activated platelets and recruits C3(H_2_O) to promote formation of cell-bound C3(H_2_O)Bb (42). This pathway supports the notion that platelets cooperate with activated leukocytes to trigger complement activation via the AP, amplifying generation of C3b, and formation of a stable C3bBb AP C3 convertase.

As noted above, the three complement pathways converge with the formation of their respective C3 convertases, and generation of C3a, and C3b (Figure 1). If activation is sufficient, excess C3b binds to these convertases, to generate C5 convertases, which cleave C5 into C5b, and C5a. C5a is the most potent anaphylatoxin, with a range of pro-inflammatory and pro-coagulant properties (see below). C5b binds to C6, and assembles with C7, C8, and multiple C9 molecules, yielding C5b-9, the so-called membrane attack complex (MAC), which has pore-forming, lytic properties designed to destroy invading organisms and damaged/foreign cells (see below) (43).

### Regulation of Complement

Complement activation is tightly regulated at multiple levels to prevent host cell damage and to allow healing to proceed. This is achieved via the coordinated actions of several membrane anchored and fluid-phase regulators, some of which will be discussed [(35); Figure 1]. C1-esterase inhibitor (C1-INH) is a serine protease inhibitor that dampens the CP and LP by neutralizing C1r, C1s, MASP-1, and MASP-2, each interaction variably potentiated by heparan sulfate and polyphosphate (37, 44, 45). It is synthesized by hepatocytes, but also by fibroblasts, endothelial cells, monocytes, megakaryocytes, and platelets. C1-INH also interferes with several pro-coagulant and pro-inflammatory enzymes, including factors XIa, XIIa, and kallikrein. Factor H (FH) is the major fluid-phase negative regulator of the AP (46). Synthesized by hepatocytes, but also by endothelial cells, platelets, and monocytes (47, 48), FH is a cofactor for factor I (FI) mediated inactivation of C3b, a decay accelerating factor of the AP C3 convertase, and a competitor to FB binding to C3b. A platelet-released kinase (49) can reduce FH binding to C3b, thereby enhancing the inflammatory response (50). FH also colocalizes with VWF in Weibel-Palade bodies and variably modulates ADAMTS13-mediated proteolysis of ultra large VWF (ULVWF) multimers, thereby impacting on platelet-vessel wall interactions (51–54), and thus atherogenesis. Negative regulators of the terminal pathway of complement activation include CD59, which binds to C8 and C9 and prevents C9 polymerization (55), clusterin and vitronectin, which reduce membrane integration of C5b-9 (56, 57), and polyphosphate, which destabilizes the C5b,6 complex, reducing C5b-9 insertion into the membrane (58).

## Platelet-Complement Cross-Talk Synergizes in Atheroma Formation

Considerable evidence supports the notion that complement and platelets act in concert to orchestrate the early cellular and molecular events that promote atheroma formation [reviewed (59, 60)]. Importantly, there exist reciprocal signaling pathways between platelets and the complement system (61) that impact on the vascular endothelium, and potentiate or attenuate each other's pro-atherogenic properties.

### Pro-Atherogenic Properties of Complement: Targeting the Vascular Endothelium

The complement system is a major contributor to inflammation and thrombosis, critical features underlying atheroma formation, and progression of atherosclerosis. Indeed, many complement components are found in atheromatous lesions where they participate in initiating and sustaining inflammation (62, 63). Endothelial cells express receptors for several complement components, most notably C1q (64), C3a (65), and C5a (66). The anaphylatoxins C3a and C5a bind to their cognate G protein-coupled receptors (C3aR for C3a; C5aR1, and C5L2 for C5a) and trigger pro-inflammatory and pro-thrombotic activities. Both C3a and C5a induce endothelial cell expression of pro-inflammatory IL-8, IL-1, and RANTES. They upregulate expression of key leukocyte adhesion molecules, VCAM-1, ELAM-1, ICAM-1, and P-selectin (67, 68). They also induce a pro-thrombotic phenotype through C5a-mediated tissue factor (TF) expression on neutrophils (69) and endothelial cells, and VWF secretion from endothelial cells (67). Indeed, C3a and C5a are pivotal in recruiting and activating monocytes, neutrophils and macrophages, promoting endothelial permeability (70), and providing a nidus for clot formation, all of which are required for the initiation, and expansion of an atherosclerotic plaque. The terminal pathway complexes, C5b-7, C5b-8, and C5b-9 also participate by augmenting VCAM-1 expression by endothelial cells (71), inducing cellular release of pro-inflammatory mediators, such as IL1-α, that cause leukocyte recruitment (72), and promote functional expression of TF. This occurs partly via activation of MAPKinases leading to its transcriptional upregulation (73), and by inducing its activation via oxidation of cell surface protein disulfide isomerase (PDI) (43, 74).

### Complement-Platelet Crosstalk

**Figure 2:**
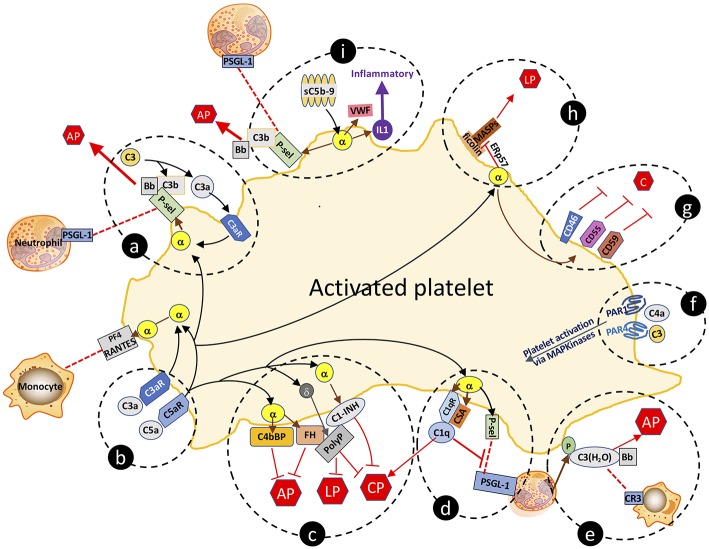
Complement-platelet interactions that can facilitate an inflammatory response that favors atheroma formation. Selected interactions between complement and platelets, as described in the manuscript, are highlighted. **(a,b)** C3a and C5a bind to their cognate receptors to trigger release of factors from α-granules. P-selectin localizes to the platelet surface and is a receptor for leukocyte expressed PSGL-1 and for C3b, the latter which allows for initiation of the AP and amplification of C3a-triggered platelet activation. **(c)** Complement activation on the platelet surface is dampened by α-granule release of cofactors C4bBP, FH and C1-INH, and δ-granule release of the anti-complement, prothrombotic polyphosphate. Polyphosphate binds to FH and C1-INH, and downregulates complement activation via the CP and the terminal pathway. **(d)** By binding to C1qR, C1q also triggers α-granule release of P-selectin and chondroitin sulfate (CSA), the latter which enhances the C1q-C1qR interaction, initiating the CP but negatively regulates leukoctye recruitment by interfering with P-selectin-PSGL-1 interactions. **(e)** Neutrophil-released properdin (P) stabilizes the convertases and is also a receptor for C3(H_2_O) which complexes with Bb to form the AP C3-convertase, and is a ligand for leukocyte-expressed CR3, thereby facilitating leukocyte cell migration to the site of inflammation. **(f)** C4a and C3 can activate platelets via distinct interactions with PAR1 and PAR4. **(g)** Activated platelets are also protected against complement-mediated destruction by granule release of negative regulators of complement, cell surface expressed CD46, CD55, and CD59. **(h)** Sublytic C5b-9 (sC5b-9) triggers platelet activation with release of VWF, P-selectin and inflammatory cytokines (e.g., IL1), the latter which further promotes inflammation. **(i)** Ficolins on the surface of activated platelets are receptors for MASPs which can trigger the LP. Release of the isomerase ERp57 modifies the ficolin to limit its functional capacity to trigger complement. C, complement activation pathways; AP, alternative pathway; LP, lectin pathway; CP, classical pathway; PF4, platelet factor 4; C3aR, C3a receptor; C5aR, C5a receptor; C1qR, C1q receptor; P-sel, P-selectin; PSGL-1, P-selectin glycoprotein ligand-1; sC5b-9, sublytic C5b-9; VWF, von Willebrand factor; α, α-granule; δ, δ-granule; ERp57, endoplasmic reticulum protein 57; PAR, protease activated receptor; P, properdin; CR3, complement receptor 3; PolyP, polyphosphate; IL1, interleukin 1.

Like endothelial cells, platelets express receptors for C1q (75), C3a (76), C4 (77), and C5a (59). They also release complement components upon activation, including C1q, C3, C4, and C5b-9 (61). These platelet-derived complement factors/complexes may promote atherogenesis in several ways. Firstly, secreted platelet-derived complement components can activate other platelets via autocrine and paracrine signaling. Secondly, as recognized in the preceding section, complement proteins from activated platelets propagate vascular inflammation by further activating the endothelium and/or recruiting leukocytes to nascent atheroma. Thirdly, and as further discussed, various complement components retained at the platelet surface can serve as substrates for continued complement activation, resulting in a tightly regulated, positive feedback loop of complement, and platelet activation (78). Several of the following described complement-platelet interactions that may impact on atherogenesis are depicted schematically in Figure 2.

The direct correlation of expression levels of receptors for C3a and C5a with platelet activation in patients with coronary artery disease supports their potential pathophysiologic relevance (59). Via their cognate receptors, C3a and C5a trigger platelet activation and aggregation (79), inducing exposure of P-selectin and the receptor for C1q (C1qR). P-selectin is a receptor for C3b, providing a site for assembly of the AP C3 convertase, and ultimately, if not checked by negative regulators, for formation of the C5b-9 MAC (80). P-selectin is also a ligand for leukocyte-expressed P-selectin glycoprotein ligand-1 (PSGL-1), which together are strongly implicated in promoting atherogenesis by enhancing recruitment of leukocytes to sites of inflammation (81).

C1q binding to C1qR cooperates with chondroitin sulfate A (CSA) that is released from α-granules of activated platelets to support C1q-mediated complement activation via the CP (82, 83). The C1q:C1qR interaction also induces P-selectin release and platelet activation, adhesion, and aggregation (84). Interestingly, C1q plays dual and apparently opposing roles in the inflammatory response, as it also dampens platelet-neutrophil aggregate formation (85) by interfering with P-selectin-PSGL-1 interactions. This is consistent with platelets possessing multiple negative regulatory mechanisms to keep inflammation in check, and to also provide protection to the platelet from complement mediated destruction. Indeed, the anaphylatoxins C3a and C5a, acting via their cognate receptors, also trigger platelet release of FH and C4b binding protein (C4bBP), both of which are negative regulators of the AP of complement (Figure 2), the latter which also negatively regulates toll like receptor (TLR)1/2 mediated pro-inflammatory cytokine production (86, 87).

Evidence from human studies supports the notion that the C3a/C3aR axis participates in atheroma formation. In a cross-sectional analysis of >500 individuals, plasma levels of C3a strongly and positively correlated with carotid artery intima-media thickness (88). In patients with coronary artery disease, there was also a strong positive correlation of C3aR expression on activated platelets with that of GpIIbIIIa, with experimental evidence that the C3a/C3aR axis regulates platelet function via activation of the small GTPase Rap1b (59, 76). In that manner, the C3a receptor (C3aR) directly impacts on hemostasis, since mice lacking C3aR exhibit prolonged bleeding times, with reduced ADP-triggered platelet aggregation. They are also protected against C3a-triggered thrombosis, with reduced severity of experimental stroke and myocardial ischemia (76).

The two structurally homologous but distinct C5a receptors, C5aR1 (CD88), and C5aR2 (C5L2), have been reported to be highly expressed in atherosclerotic lesions (6). Blockade or gene inactivation in mice of either of these, results in protection against diet-induced atherosclerosis (89), consistent with what is observed when C5a is blocked (90). Most intriguing, blockade of both C5aR1 and C5aR2 had added benefit in reducing neointimal plaque size and inflammation in a wire injury model. Since the C5a receptors are widely expressed by many cell types, the specific role of the receptors on platelets was not determined. Nonetheless, the findings are impressive, and imply that a multi-modal approach to tackle atherosclerosis will likely yield improved outcomes.

C4a, generally accepted to be the weakest of the three anaphylatoxins, has unique properties in terms of crosstalk with other innate immune pathways to induce platelet activation. Although a specific receptor for C4a has remained elusive, recent studies indicate that C4a binds to PAR1 and PAR4 as an untethered agonist, triggering activation of downstream pro-inflammatory MAPKinases, thereby participating in platelet activation via alternative routes (91). The pathophysiologic relevance of this pathway in atherogenesis has not yet been explored.

C3 itself also plays an important role in platelet function. C3-deficient mice exhibit delayed hemostasis, based on tail bleeding times and are protected against atheroma formation in the atherosclerosis-prone low density lipoprotein receptor-null (*Ldlr*^−/−^) mice (92). Aggregation of the C3-deficient platelets was significantly dampened in response to the PAR-4 agonist peptide (93), a defect that was rescued by the addition of exogenous plasma C3 [(93), Figure 2]. Consistent with these findings, C3-null platelets stimulated with convulxin (an agonist for glycoprotein VI, GPVI), respond with reduced surface exposure of P-selectin, von Willebrand factor (VWF), and annexin V (94), effects that dampen both inflammation and hemostasis/thrombosis. As mentioned previously, C3 is readily hydrolyzed to C3(H_2_O) which may bind to the activated platelet surface in the presence of leukocyte derived properdin, promoting formation of a platelet surface-bound C3(H_2_O)Bb convertase (42). This would thus enable complement activation to proceed via the AP. When associated with the platelet, C3(H_2_O) may also serve as a ligand for leukocyte cell surface receptor CD11b/CD18 [also referred to as complement receptor 3 (CR3) or integrin α_M_β_2_], facilitating formation of platelet-leukocyte interactions and recruitment of activated macrophages to atheroma. Again, the physiologic relevance of each of these independent interactions remain unclear, but taken together support the *in vivo* evidence of a role for C3/C3(H_2_O) in atherosclerosis (92).

In contrast to the strong evidence that C3, C3a/C3aR, and C5a/C5aR are important contributors to platelet activation and atherogenesis, the role of C5 in modulating platelet function in health and vascular disease is more controversial. This is in spite of most studies concluding that C5 participates in promoting tissue factor mediated fibrin clot formation *in vivo*. In murine models, histones trigger aggregation of platelets from wild-type mice but not from C5-deficient mice (95), suggesting that C5 is essential for normal platelet function. These findings however, seem in conflict with the work of others, in which platelets from C5-deficient mice responded normally to agonist induced platelet release of P-selectin, vWF, and annexin V, and furthermore, did not exhibit any hemostatic or vessel wall platelet deposition defects *in vivo* (94). Moreover, the development of atherosclerosis in *ApoE*(-/-) mice was not affected by genetic deletion of C5 (96). Such apparent discrepancies in the interpretation of the role of C5 in platelet function and vascular disease highlight the need for further study.

The lectin pathway (LP) also participates in platelet activation and function (97). Ficolins (but not MBL), MASP-1, and MASP-2, were detected as complexes on the surface of activated platelets, indicating that the initiating factors can assemble to trigger cleavage of C4 and C2 (Figure 2). Moreover, ficolins and MBL were present in the plaques of atherosclerotic lesions derived from patients undergoing carotid endarterectomy (98). This pathway appears to be dampened on the cell surface by the release from activated platelets of C1-INH (Figure 2). Recent data indicate that the LP can also be attenuated by the release from activated platelets of the thiol isomerase ERp57, which interferes with ficolin recognition via disruption of its multimerization (99). While not proven, these pathways may reasonably impact on the inflammatory response to injury that leads to atheroma formation. Further *in vivo* studies will be required for validation and to ascertain whether there are targetable steps for treatment design.

The concept of platelet-released enzymes, as described above for ERp57, was reported >20 years ago (49), but is now a re-emerging area of interest. Protein kinases released from the α-granules of activated platelets in concert with ATP and divalent cations (Ca^2+^) from dense granules, have been shown to phosphorylate plasma proteins, including coagulation factors XI, Va, and protein S, thereby modifying their functions. In the context of complement, C3 phosphorylation occurs by this method, resulting in the C3b cleavage fragment being more resistant to factor I-mediated inactivation to iC3b. Degradation of the kinase-modified C3 also generates C3d that binds more avidly to complement receptor 1 (CR1;CD35), thereby enhancing opsonin activity (50) and clearance of immune complexes by phagocytosis. Genetic variations of the CR1 gene have been linked to the risk of incident coronary artery disease and inflammation by unknown mechanisms. Although entirely speculative, it is possible that the kinase-mediated modification of C3 from activated platelets may contribute.

The terminal pathway of complement also participates in platelet activation and atherogenesis. Sublytic concentrations of C5b-9 bind to the platelet surface and induce activation and α-granule secretion (79, 100). C5b-9 also induces changes in the orientation of the phospholipid membranes of platelets that favor binding of factor Va, prothrombinase assembly, and generation of thrombin (74, 101, 102). Similar to C3a and C5a, C5b-9 triggers platelet secretion of VWF, P-selectin and pro-inflammatory cytokines (e.g., IL1) (Figure 2), the expression of adhesion molecules on platelets (103, 104), and the release of platelet microparticles (PMPs), any of which may modulate vascular responses to thrombo-inflammatory stimuli.

### Self-Preservation of the Activated Platelet by Complement Regulators

The profound changes in the structure and the expression pattern of proteins and glycolipids on the surface of the activated platelet, would be expected to trigger a host innate immune response that would destroy the cell, rendering it unable to complete its prothrombotic/prohemostatic function in the setting of injury and bleeding. Yet, in spite of the platelet being activated by several complement factors (e.g., C3a, C5a, C5b-9), and providing sites for assembly of the convertases (e.g., P-selectin, CSA, C1qR), complete formation and integration of a lytic C5b-9 MAC, is normally held in check, preserving the integrity of the prothrombotic platelet. This is achieved through the action of numerous negative regulators of complement, stored in the platelet and released upon activation. Thus, platelet α-granules contain C1-INH, FH, CD55, CD59, CD46, and clusterin, while polyphosphate is housed in dense granules (105). All can accumulate on the activated platelet surface and prevent generation of the C5b-9 MAC via the CP, the LP and/or the terminal pathway (Figure 2). Indeed, we have shown that polyphosphate directly interacts with C1-INH, augmenting the serine protease inhibitor's anticomplement activity, while retaining the prothrombotic properties of polyphosphate (106), and thus, presumably, the prothrombotic function of the platelet (45, 58). Although the physiologic relevance of the platelet pool of these negative regulators in atherogenesis, has not been specifically validated, global blockade via gene inactivation or pharmacologic interventions have established their importance. Thus, for example, lack of CD55 or CD59 in atherosclerosis-susceptible *ApoE*^−/−^ mice, resulted in worse disease, while CD59 administration reduced the severity of experimental atherosclerosis by abrogating MAC formation (107, 108). Similarly, atheroma formation in *Ldlr*^−/−^ mice, was attenuated by administration of C1-INH (109).

These complement regulatory factors and a soluble form of C5b-9 also are found in/on PMPs that are released in the setting of platelet activation. The function of these PMPs is not clear, as they variably contain other complement activating factors that are found in and on platelets (60, 78, 110, 111). The PMPs also contain/express variable amounts of tissue factor, factor V, other coagulation-related factors, growth factors, cytokines, lipids, ions, and microRNAs [reviewed in (112)]. Given the complex nature of PMPs and their variability in composition under different pathological conditions, it has been challenging to discern their primary roles in different disease states and stages. Nonetheless, speculation abounds, including the notion that PMPs serve to clear sublytic C5b-9 away from the platelet (60), thereby providing local protection to the platelet.

## Conclusion

In this brief review, we highlight the complexity of the cross-talk pathways between platelets and the complement system, particularly as they pertain to early stages of atherosclerosis. As is evident, there are multiple apparently opposing factors and pathways that may promote or prevent inflammation and atheroma formation. In the delicate balance that maintains immune and vascular homeostasis in the face of multiple stresses, this is as expected. When this balance is unfavorably tipped due to genetic, epigenetic and/or environmental factors, inflammation, and atherogenesis may proceed. By understanding which factors are tipping that balance and how they function, more effective preventative and therapeutic strategies may be designed.

Notably, and in spite of promising data that complement activation directly correlates with atheroma formation and atherosclerosis, and that interfering with the complement cascade may be beneficial, at least in preclinical models, anti-complement therapies have not entered the clinic. This may be because the mouse model does not fully recapitulate the human condition. It may reflect the current high cost of the very few anti-complement drugs that are available for clinical use (e.g., eculizumab). Or it may be that appropriate trials have yet to be performed. From the complement cascade, it is evident that there are multiple potential steps at which interventions might be envisaged, that could potentially dampen feed-forward loops in/on the platelet that drive inflammation and atherogenesis. Indeed, strategic initiatives by industry and academia that aim to target the complement system to treat a range of vascular and inflammatory diseases abound. A full discussion of drugs at various stages of development is beyond the scope of this brief report. However, several of the opportunities and challenges are well-addressed in recent reviews (113–117). From the relatively simple scheme that we offer in Figure 2, treatments could include, for example, agents that interfere with C1q interactions with C1qR and/or CSA to dampen activation of the CP and reduce monocyte adhesion to endothelial cells. Antibodies against C1s are being used successfully to suppress CP-triggered cold-agglutinin disease (118) and might reasonably have efficacy in preventing atheroma formation. Cp40 is a cyclic peptide and analog of compstatin that inhibits C3-mediated activation of endothelial cells to reduce leukoctye adhesion (119). Rapamycin upregulates expression of CD55 by inducing protein kinase Cα, AMP-activated kinase, and CREB-dependent pathways, thereby dampening allograft vasculopathy, a benefit that synergizes with statin therapy (120). With experimental evidence of a role for the LP in atherogenesis, MASP-2 inhibitors that are currently being evaluated for hereditary angioedema and other disorders (121), could also be considered to intervene in the platelet-complement crosstalk driving atherogenesis.

Indeed, the near future will likely see many complement-targeted therapies enter the clinic for various innate immune/inflammatory disorders. The challenges will be how to select which ones are best for intervening in atheroma formation, whether multiple pathways should be targeted, how to select when to administer, how long, how much, and how to monitor (122). No matter which anti-complement interventions are used, effective treatments will undoubtedly require maneuvers to mitigate against the critical dietary, environmental and epi/genetic triggers that drive the disease (123).

## Author Contributions

All authors listed have made a substantial, direct and intellectual contribution to the work, and approved it for publication.

### Conflict of Interest Statement

The authors declare that the research was conducted in the absence of any commercial or financial relationships that could be construed as a potential conflict of interest.
